# Response of *Thymus lotocephalus* In Vitro Cultures to Drought Stress and Role of Green Extracts in Cosmetics

**DOI:** 10.3390/antiox11081475

**Published:** 2022-07-28

**Authors:** Inês Mansinhos, Sandra Gonçalves, Raquel Rodríguez-Solana, Hugo Duarte, José Luis Ordóñez-Díaz, José Manuel Moreno-Rojas, Anabela Romano

**Affiliations:** 1 MED–Mediterranean Institute for Agriculture, Environment and Development & CHANGE–Global Change and Sustainability Institute, Faculdade de Ciências e Tecnologia, Universidade do Algarve, Campus de Gambelas, 8005-139 Faro, Portugal; ifmansinhos@ualg.pt (I.M.); raquel.rodriguez.solana@juntadeandalucia.es (R.R.-S.); hmduarte@ualg.pt (H.D.); 2 Department of Agroindustry and Food Quality, Andalusian Institute of Agricultural and Fisheries Research and Training (IFAPA), Avenida Menendez-Pidal, SN, 14004 Córdoba, Spain; josel.ordonez@juntadeandalucia.es (J.L.O.-D.); josem.moreno.rojas@juntadeandalucia.es (J.M.M.-R.)

**Keywords:** aromatic plant, abiotic stress, green extract, phenolics, tyrosinase inhibition, UV protecting extracts

## Abstract

The impact of drought stress induced by polyethylene glycol (PEG) on morphological, physiological, (bio)chemical, and biological characteristics of *Thymus lotocephalus* López and Morales shoot cultures have been investigated, as well as the potential of iron oxide nanoparticles, salicylic acid, and methyl jasmonate (MeJA) as alleviating drought stress agents. Results showed that PEG caused oxidative stress in a dose-dependent manner, raising H_2_O_2_ levels and reducing shoots’ growth, photosynthetic pigment contents, and phenolic compounds production, especially phenolic acids, including the major compound rosmarinic acid. Moreover, Fourier Transform Infrared Spectra analysis revealed that PEG treatment caused changes in shoots’ composition, enhancing terpenoids biosynthesis. PEG also decreased the biological activities (antioxidant, anti-tyrosinase, and photoprotective) of the eco-friendly extracts obtained with a Natural Deep Eutectic Solvent. MeJA was the most efficient agent in protecting cells from oxidative damage caused by drought, by improving the biosynthesis of phenolics, like methyl 6-O-galloyl-β-D-glucopyranoside and salvianolic acids, as well as improving the extracts’ antioxidant activity. Altogether, the obtained results demonstrated a negative impact of PEG on *T. lotocephalus* shoots and an effective role of MeJA as a mitigating agent of drought stress. Additionally, extracts showed a good potential to be used in the cosmetics industry as skincare products.

## 1. Introduction

Plants face abiotic stresses every day, being affected at several levels, such as morphological, physiological, biochemical, and metabolic. According to climate change models, the Mediterranean basin is one of the regions across the globe that will be strongly affected by this situation, especially by drought events [1]. Drought is one of the biggest concerns that delimits plant development, causing severe or lethal consequences. Water comprises 80–90% of the biomass of plants and is implicated in many critical physiological functions, such as growth and photosynthesis [2,3]. Usually, drought stress triggers the accumulation of reactive oxygen species (ROS), which act as signaling molecules, leading to changes in several stress-responsive genes expression [4] and in the biosynthesis of secondary metabolites [5]. Furthermore, the accumulation of osmolytes, such as proline, soluble sugars, and betaines is crucial to maintain the cell water status and alleviate the oxidative damage caused by water deficiency [4]. Typically, tolerant plants exhibit a significantly lesser number of metabolites accumulation than sensitive plants, though demonstrating an increment in osmolytes production [2]. Nevertheless, the plant’s capacity to survive under stressed conditions depends on several factors, such as plant species, growth stage, and extent and intensity of the stress conditions. In vitro simulation of abiotic stresses is an advantageous approach to investigating plants’ response to those factors. Besides being performed under controlled conditions and free of pathogens, in vitro culture allow the production of plant material on a large scale, without compromising species conservation. Moreover, in vitro culture allows the application of several strategies to change the production of secondary metabolites (e.g., adding precursors or elicitors to the culture media) [6]. Among osmotic agents that can be applied to mimic drought conditions in vitro, polyethylene glycol (PEG) is the most commonly used. PEG increases the solute potential of the culture media, blocking the absorption of water by the plant [7]. Moreover, owing to its high molecular weight, this osmotic agent can decrease the water potential without being phytotoxic or taken up by the plant [4]. Different agents, such as iron oxide (Fe_3_O_4_) nanoparticles (NPs), salicylic acid (SA), and methyl jasmonate (MeJA) were tested for their capacity to alleviate the deleterious impact of drought stress. Included in abiotic nano compounds, Fe_3_O_4_ NPs have been shown to stimulate several mechanisms in stressed plants, as well as to improve the biosynthesis of secondary metabolites [8]. SA and MeJA are two important endogenous plant growth regulators involved in stress tolerance in several plant species [9], which, when applied exogenously, have a great capability as elicitors [10].

The genus *Thymus* is the eighth-most abundant genus of the Lamiaceae (Labiatae) family [11], comprising about 350 worldwide species of perennial, aromatic herbs, and subshrubs [7,12]. These perennial herbs have been known since ancient times in virtue of their medicinal and aromatic attributes [2,11], being widely used as flavoring agents, culinary herbs, and ornamentals [6]. More recently, *Thymus* have been demonstrated to exhibit a broad range of functional opportunities for food, pharmaceutical, and the cosmetic industries [13], due to their main groups of secondary metabolites–volatile and phenolic compounds–being responsible for a great diversity of biological actions [12]. *Thymus lotocephalus* López and Morales is a Mediterranean species endemic to the Algarve, a southern region of Portugal, that is listed as Near Threatened in The IUCN Red List of Threatened Species [14]. Previous studies indicated that this species produces phenolic (e.g., rosmarinic acid, salvianolic acids, luteolin, caffeic acid) and volatile (e.g., linalool, caryophyllene oxide, camphor, borneol) compounds with biological effects, and that in vitro culture is a good alternative for the production of bioactive compounds from this species, without compromising natural populations [6,15,16,17]. Phenolic compounds, which may be categorized as phenolic acids, flavonoids, tannins, stilbenes, lignans, and coumarins, present a great potential for therapeutic applications to numerous human diseases, such as diabetes, cancer, neurodegenerative and cardiovascular pathologies, due to their multifunctional biological activities, particularly antioxidant [18].

The first decade of the 21st century was marked by the significant growth in sales of cosmetic products, representing 23% of the total market share of consumer products. The consumer demand for innovative and natural products to treat skin is continuously increasing. The harmful effects on human health of excessive exposure to UV (ultraviolet) photons (UV-A and UV-B) are well known and, under climate change context, the correct use of products protecting the skin is even more essential. UV filters can be classified into organic (natural or artificial) and inorganic substances. In general, while inorganic compounds reflect and scatter UV radiation, organic sunscreens absorb it [19]. The safety and efficacy of most artificial sunscreens ingredients are hampered by their photostability, toxicity, and damage to aquatic ecosystems [20], so new strategies are being looked for and developed. One increasingly appreciated strategy consists of the application of plant extracts (or their isolated bioactive compounds) as ingredients, instead of synthetic chemicals. Phytochemicals exhibit antioxidant activity, which is very important to fight against environmental free radicals caused, for example, by UV radiation, which trigger skin-damaging conditions. In addition, antioxidants are essential in several cosmetic formulations, such as facial anti-aging, antioxidant-based, anti-allergy, and dry skin hydrating products [21]. Besides the other recognized biological properties of phytochemicals, which are extremely valuable to cosmetic products, such as antioxidant, anti-inflammatory, anti-aging, and antimicrobial, they also present the ability to absorb the energy of photons, making them potential UV-protecting agents [22,23]. In this scenario, plant extracts, or their isolated bioactive compounds, can be used in sunscreen formulations. Aging signs (e.g., wrinkles, hyperpigmentation, flaccidity, texture changes) are another concern of modern society and therefore the demand for anti-aging products is, likewise, increasing. Melanogenesis is the process responsible for melanin production as a defensive mechanism against UV radiation, in which tyrosinase is a key enzyme. The excessive accumulation of melanin in the skin can cause hyperpigmentation, so the inhibition of tyrosinase is a target in the cosmetic industry [24].

The solvent used to extract plant bioactive compounds for cosmetic applications is of utmost importance. Ethanol and glycols are two conventional solvents largely employed to extract polar or semi-polar compounds, but they present some drawbacks. In addition to ethanol requiring specific safety procedures due to its volatility and flammability nature, it can also disturb the skin hydrolipidic film and irritate sensitive skins due to its drying nature. Glycols are usually associated with petrochemicals and, for this reason, consumers often try to avoid glycol-containing cosmetic formulations. Water is also used to extract polar compounds; however, microbiological safety is its major limitation. Recently, Natural Deep Eutectic Solvents (NADES) have appeared as new eco-friendly alternatives to conventional solvents, which are recognized to be dangerous to human health and the environment. NADES are composed of at least two components, a hydrogen bond acceptor (HBA) and a hydrogen bond donor (HBD), having a much lower melting point than that of their individual components [18]. Due to their natural origin, biodegradable and non-toxic nature, NADES represent an unexplored opportunity to develop innovative extracts with unique phytochemical footprints and biological properties, making it possible to be securely employed in the pharmaceutical, food, and cosmetic industries [18,25]. In the previous study conducted by our group, NADES were demonstrated to be more effective than conventional solvents (methanol, ethanol 80%, and water) to extract phenolic compounds from a Lamiaceae species [18].We hypothesize that, as in other Mediterranean species, drought stress might have harmful effects on *T. lotocephalus* and that the use of some agents may alleviate these negative effects. In this context, the key goal of this work is to investigate how drought induced by PEG (2, 5, and 7%) influences some morphological, physiological, and (bio)chemical traits *of T. lotocephalus* shoot cultures, as well the biological activities (antioxidant, anti-tyrosinase, and photoprotective) of extracts obtained with NADES. The potential of Fe_3_O_4_ NPs, SA, and MeJA in alleviating drought stress effects were also investigated. As far as we know, this is the first report investigating the response of *T. lotocephalus* to drought stress, analyzing the chemical structures of this species using FTIR spectroscopy and using NADES for the extraction of their bioactive compounds. In a climate change scenario, these studies are important as a first approach to understanding the response of *T. lotocephalus* to drought stress, and how these conditions can affect the potential applications of this aromatic species. Results from this study can also be useful to predict the impact of drought stress conditions on other Mediterranean aromatic species with medicinal/industrial properties.

## 2. Materials and Methods

### 2.1. Chemicals and Reagents

L-Proline (>99%), PEG 6000, ninhydrin, 2,2′-azobis(2 methylpropionamidine)dihydrochloride (AAPH), (±)-6-hydroxy-2,5,7,8-tetramethylchromane-2-carboxylic acid (Trolox) and potassium ferricyanide (K_3_[Fe(CN)_6_]) were acquired from Acros Organics (Geel, Germany). Lactic acid, and fluorescein were purchased from Panreac (Barcelona, Spain). Sodium carbonate anhydrous (Na_2_CO_3_), ferric chloride (FeCl_3_), Folin-Ciocalteu’s reagent, and gallic acid were acquired from VWR (Leuven, Belgium). Ascorbic acid, acetic acid, and potassium iodide were acquired from Merck (Darmstadt, Germany). Salicylic acid (SA), methyl jasmonate (MeJA), Fe_3_O_4_ nanoparticles (50–100 nm), hydrogen peroxide (H_2_O_2_), trichloroacetic acid (TCA), L- proline (≥99%), 2,2-diphenyl-1-picrylhydrazyl (DPPH), 3,4-dihydroxy-L-phenylalanine (L-DOPA), 2,2′-azino-bis (3-ethylbenzothiazoline-6-sulfonic acid) diammonium salt tablets (ABTS), mushroom tyrosinase (EC 1.14.18.1), kojic acid, potassium persulfate (K_2_S_2_O_8_), potassium bromide, HPLC-MS-grade water, HPLC-MS-grade acetonitrile, luteolin, epigallocatechin gallate, protocatechuic acid, and formic acid were obtained from Sigma–Aldrich (Steinheim, Germany). Rosmarinic acid and quercetin were supplied by Extrasynthese (Genay, France), and ρ-coumaric, caffeic acid, and catechin were provided by AASC Ltd. (Southhampton, UK).

### 2.2. Plant Material, In Vitro Culture Conditions, and Experiments

Shoots of *T. lotocephalus* were proliferated in vitro as described by Coelho et al. [26] in MS medium [27], containing 2% (*w*/*v*) sucrose and 0.7% (*w*/*v*) agar, and pH was corrected to 5.75 ± 0.05. Culture media were autoclaved for 20 min at 121 °C. To induce drought, different concentrations of PEG 6000 [2, 5, or 7% (*w*/*v*)] were added to the culture media, according to the diffusion-based method reported by Girma and Krieg [28]. PEG was spread on the MS solidified medium surface for 24 h. The concentration of PEG that induced higher drought effects, 7%, was selected to evaluate the potential of Fe_3_O_4_ NPs, SA, and MeJA in alleviating drought stress effects. Thus, these agents were added to PEG-free and 7% PEG culture media. Fe_3_O_4_ NPs (10 mg/L) were added before autoclaving, and SA (50 µM) and MeJA (50 µM) solutions were sterilized through 0.2 μm microfilters before addition to the autoclaved media. Multiplication medium was used as control. Cultures were incubated at 25 ± 1 °C, with 16 h light (40 μmol m^−2^ s^−1^, cool white fluorescent lamps) and 8 h dark cycle, for 7 weeks. Twelve Erlenmeyer flasks with seven shoots each were tested for each treatment.

### 2.3. Morphological Traits

After culture for 7 weeks, the biometric parameters (total number of shoots, the longest shoot length, and the fresh and dry weight of the biomass) were registered. Shoots were dried at 40 °C until constant weight to determine the dry weight.

### 2.4. Physiological and Biochemical Measurements

Physiological attributes such as the chlorophyll and carotenoids, hydrogen peroxide (H_2_O_2_), and proline contents were assessed using fresh material. Chlorophylls and carotenoids were extracted, according to Lichtenthaler [29], using pure acetone and 25 mg of plant material. The absorbance was measured at 661.6, 644.8, and 470 nm using UV–Vis spectrophotometer (T70+ UV/Vis Spectrophotometer, PG Instruments Ltd., Leicestershire, UK).

The H_2_O_2_ content was determined according to a method from Loreto and Velikova [30] with slight alterations. A hundred milligrams of plant material were ground in ice with 0.1% TCA. The same volume of the supernatant obtained after centrifugation (15 min at 12,000× *g*) and 10 mM potassium phosphate buffer were mixed, before adding the double 1 M KI solution. The absorbance was measured at 390 nm (Tecan Infinite M200 microplate reader, Männedorf, Switzerland) after 30 min in darkness. For proline estimation, plant material was extracted three times using 80% (*v*/*v*) ethanol at 80 °C for 30 min [31]. Then, the extract was incubated for 1 h at 100 °C with 1% (*w*/*v*) ninhydrin reagent prepared with 60% (*v*/*v*) acetic acid. After cooling, and the addition of toluene, the absorbance of the organic phase was recorded at 520 nm.

### 2.5. Fourier Transform Infrared Spectra (FTIR) Spectroscopy

Functional group distribution of the main compounds present in samples was followed by FTIR spectroscopy (Bruker Tensor 27, Billerica, MA, USA). For that, the samples were dried at 60 °C, mixed with potassium bromide, pressed into KBr pellets, and measured in a 4000–600 cm^−1^ range. Each FTIR spectra was evaluated by the data analysis and graphing software OriginPro, version 2022 (OriginLab Corporation, Northampton, MA, USA) and compared with those of other reports [32,33,34,35,36].

### 2.6. Extraction of Phenolic Compounds

The plant material was dried at 40 °C until constant weight and powdered to <2 mm. A green extraction was performed using proline: lactic acid (1:1) mixture containing 30% (*w*/*w*) water as NADES according to Mansinhos et al. [18], using a plant/solvent ratio of 2.5:100 (*w*/*v*). Extraction was performed in an ultrasound bath (Elmasonic S 100 (H, Elma Hans Schmidbauer GmbH & Co. KG, Singen, Germany) using a frequency of 37 kHz for 30 min at 50 °C. After being filtered using a Whatman n°. 1 filter paper (Whatman Int. Ltd., Maidstone, UK), the extracts were kept at −20 °C up to usage.

### 2.7. Spectrophotometric and Chromatographic Analysis for Phenolic Compounds

#### 2.7.1. Spectrophotometric Measurement of Total Phenolic Content (TPC)

The content of total phenolics in the extracts was determined using Folin-Ciocalteu (F-C) reagent, as described by Ainsworth and Gillespie [37]. A mixture containing 200 μL 10% (*v*/*v*) F-C reagent, 100 μL plant extracts diluted in phosphate buffer (75 mM, pH 7.0), and 800 μL Na_2_CO_3_ (700 mM) was incubated for 2 h. The standard used was gallic acid (0.004–0.5 mM) and the absorbance was recorded at 765 nm. The results were calculated as gallic acid equivalents per gram of dry weight (mg_GAE_/g_DW_).

#### 2.7.2. Analysis of Individual Phenolic Compounds by HPLC-HRMS

The plant extracts were examined utilizing a Dionex Ultimate 3000 HPLC system, with an HPLC pump and an autosampler operating at 10 °C (Thermo Fisher Scientific, San Jose, CA, USA). The sample separation was performed on a 150 × 4.6 mm i.d. 5 μm 100 A C18 Kinetex column (Phenomenex, UK) with 1 mL/min of flow rate and with 40 °C of column temperature. The chromatographic conditions were performed according to Gonçalves et al. and Mansinhos et al. [17,18]. The solvent system was composed of solvent A (distilled water) and solvent B (acetonitrile), both with 0.1% formic acid. Gradient mode was 0 min—90% A; 10 min—74% A; 22 min—35% A; 30 min—5% A; 40 min—5% A; 40.1 min—90% A; and 45 min—90% A. The column flow rate was 0.2 mL/min directed to an Exactive Orbitrap mass spectrometer (Thermo Fisher Scientific, San Jose, CA, USA) fitted with a heated electrospray ionization probe (HESI). Negative ions were analyzed at scan mode of auto MS/MS, at the range of 100–1000 *m*/*z*. The analyses were also based on in-source collision-induced dissociation scans at 25 eV. The source condition was the spray voltage of 4000 V, the capillary temperature of 320 °C, heater temperature of 150 °C, and the sheath gas and auxiliary gas flow rate of 25 and 5 units, respectively. Identification of compounds was based on the retention time and the exact mass in conjunction with standards. When standards were unavailable, it was compared with the theoretical exact mass of the molecular ion with the determined accurate mass of the molecular ion, to tentatively identify the compound and was looked for in several metabolite databases (PubChem, Metlin, ChemSpider, Phenol Explorer). In addition, the biocompound’s identification was performed following the MSI MS levels [38]. Supplementary Table S1 summarizes the chemical formula, theoretical exact mass, delta ppm, retention time (RT), and MSI MI level of the compounds. To quantify the compounds, the theoretical exact mass of the molecular ion was selected by standard curves, or by the calibration curve of a close parent metabolite based on the structure. Limits of detection (LOD) and quantification (LOQ) were determined from the standard deviation of ten blank determinations, ranging LOD and LOQ from 0.10 to 228.13 µg/L and 0.33 to 760.43 µg/L, respectively. The criteria used in the quantification of phenolics are outlined in Supplementary Table S2. The results were expressed in milligrams per kilogram of extract.

### 2.8. Assessment of the Biological Properties of the Extracts for Dermo-Cosmetic Application

#### 2.8.1. Antioxidant Activity

The antioxidant activity of the extracts was assessed using different assays with two mechanisms [atom hydrogen transfer-based method (ORAC), single electron transfer-based method (FRAP), and mixed methods making use of hydrogen-atom transfer and single-electron transfer (DPPH and ABTS)].

##### DPPH Free Radical Scavenging

Applying the method described by Soler-Rivas et al. [39], the capacity of the plant samples to scavenge the free radical DPPH^•^ was analyzed. For that, before 30 min of incubation, 30 µL extract was mixed with 300 μL DPPH methanolic solution (90 μM) and methanol 80% until 900 μL. The absorbance was measured at 515 nm, using Trolox (0.025–0.3 mM) as standard, and the results were expressed as milligrams of Trolox equivalents per gram of dry weight (mg_TE_/g_DW_).

##### ABTS Free Radical Scavenging

The ABTS^•^ free radical scavenging activity of the extracts was analyzed according to Re et al. [40]. Using K_2_S_2_O_8_, the ABTS stock solution (7 mM) was prepared and stored for 12–16 h. After this period, the stock solution was diluted with H_2_O until obtaining an absorbance (734 nm) of 0.700 ± 0.02. The samples (10 µL) were added to the diluted ABTS solution (190 µL) and the absorbance was read, using Trolox as standard. The results were expressed as milligrams of Trolox equivalents per gram of dry weight (mg_TE_/g_DW_).

##### Ferric Reducing Antioxidant Power (FRAP)

FRAP assay comprises the reduction of Fe (III) to Fe (II) in the presence of an antioxidant. Based on the method described by Yen and Chen [41], the reaction involving the plant extracts (100 μL), 1% K_3_[Fe (CN)_6_] (250 μL), and potassium phosphate buffer (200 mM, pH 6.6) (250 μL) was incubated for 20 min at 50 °C. After it was added 10% TCA (250 μL) to the reaction and centrifuged. FeCl_3_ (80 μL) at 0.1% was mixed with the same amount of supernatant and water (400 μL). The absorbance was measured at 700 nm, using ascorbic acid as standard, and the results were expressed as milligrams of ascorbic acid equivalents per gram of dry weight (mg_AAE_/g_DW_).

##### Oxygen Radical Absorbance Capacity (ORAC)

According to Gillespie et al. [42], plant extracts (25 μL) were incubated with fluorescein (0.2 μM) for 10 min at 37 °C. Then, 150 mM AAPH (25 μL) was added and the fluorescence was read every 5 min (90 min), up to value zero at 530 nm emission and 485 nm excitation. Applying the differences in areas under the fluorescein decay curve between the blank and the samples, the results were calculated. Trolox was used as standard and the results were expressed as milligrams of Trolox equivalents per gram of dry weight (mg_TE_/g_DW_).

#### 2.8.2. Inhibition Effects against Melanogenesis Key Enzyme

The tyrosinase (Tyr) inhibitory assay was carried out according to Masuda et al. [43]. The extracts (50 μL) were mixed with mushroom Tyr (50 μL, 46 U/mL) and 20 mM sodium phosphate buffer (80 μL, pH 6.8), and incubated for 10 min at room temperature. After the addition of 80 μL of the substrate (L-DOPA, 2.5 mM) and an incubation period of 10 min at room temperature, the absorbance was recorded at 475 nm (microplate reader). The results were calculated as kojic acid equivalents (mg_KAE_/g_DW_).

#### 2.8.3. Photoprotective Properties

The capacity of the plant extracts as a natural filter protecting from ultraviolet (UV) radiation was examined using UV–Vis spectrophotometer. UV spectra of the extracts at 250 μL/mL were measured in the range from 250 to 400 nm with an interval of 2 nm. The extraction solvent [proline: lactic acid (1:1) with 30% (*w*/*w*) water] was used as a blank.

#### 2.8.4. Determination of Sun Protection Factor (SPF)

The photoprotective activity of the extracts was evaluated by measuring the SPF values, which are frequently utilized to evaluate the efficacy of sunscreen against UV radiation. Extracts were diluted in the extraction solvent to obtain different concentrations (50–2500 μL/mL), and the present solvent served as a blank. Spectrophotometric scanning was accomplished at wavelengths in the range of 290–320 nm, with intervals of 5 nm with UV–Vis spectrophotometer. SPF values were obtained following the equation developed by Mansur et al. [44]:

$$\mathrm{SPF}=\mathrm{CF}\times \overset{320}{\underset{290}{\sum }}\mathrm{EE}\left(\lambda \right)\times I\left(\lambda \right)\times \mathrm{Abs}\left(\lambda \right)$$

where CF is the correction factor (=10); EE(λ) is the erythemal effect spectrum; I(λ) is the solar intensity spectrum; Abs(λ) is the absorbance. The values of EE(λ) × I(λ), described by Sayre et al. [45] are presented in Table S3.

### 2.9. Statistical Analysis

Data are presented as mean ± standard error for the total number of the results and analyzed by one-way analysis of variance (ANOVA), and Tukey’s New Multiple Range Test (*p* < 0.05). Correlations were determined using Pearson’s test. Statistical analyses were performed by IBM SPSS Statistics for Windows (version 26.0, Armonk, NY, USA: IBM Corporation). Hierarchical cluster analysis, K-means cluster analysis, and Principal Component Analysis (PCA) were analyzed by the software OriginPro, version 2022 (OriginLab Corporation, Northampton, MA, USA).

## 3. Results and Discussion

### 3.1. Evaluation of Shoots Growth

**Figure 1:**
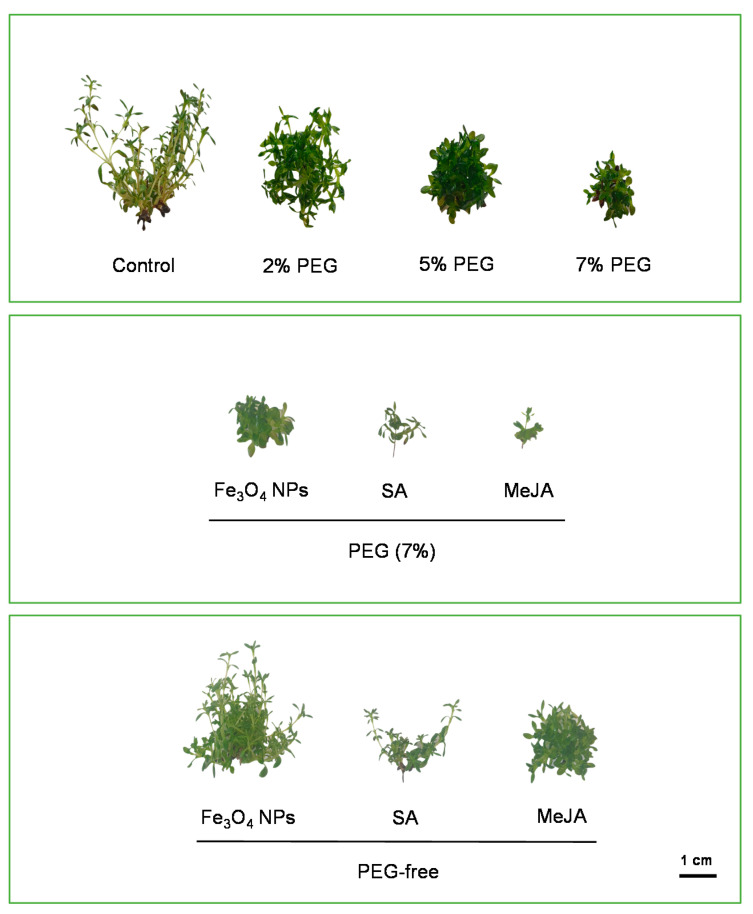
The aspect of in vitro shoots of *Thymus lotocephalus* grown in media with 0 (control), 2, 5, or 7% of PEG, Fe_3_O_4_ nanoparticles (NPs), salicylic acid (SA), methyl jasmonate (MeJA), 7% PEG + Fe_3_O_4_ NPs, 7% PEG + SA, or 7% PEG + MeJA after 7 weeks of culture. The scale bar represents 1 cm.

Physiological mechanisms of plants, especially growth and development, are sensitive to water limitations. The visual appearance of *T. lotocephalus* in vitro-regenerated shoots cultured in different media is shown in Figure 1. Results show that the increasing PEG concentration gradually reduced the mean shoot length. The highest PEG concentration (7%) caused the biggest decrease in shoot length (11.5 ± 0.43 mm), which was statistically different from that of the control (48.3 ± 2.68 mm) and the PEG concentration 2% (Table 1). Similar results were obtained in in vitro cultures of *Thymus vulgaris* [4], *Thymus citriodorus* [7], *Salvia leriifolia* [46], *Stevia rebaudiana* [3], and *Amsonia orientalis* [47], under drought stress. When plants are subjected to drought stress, the water movement through the xylem decreases, and to maintain turgor status plant cells have to reduce their osmotic potential [47]. The decrease in shoot length could be explained by the restricted water absorption and the turgescence pressure for cell enlargement, which disrupt cell division and elongation [46]. Curiously, in comparison with the control, the shoot number and the biomass produced were higher in PEG-containing media, especially with 2%. Even so, shoots obtained in the control group demonstrated normal aspect (Figure 1), while those produced in media with PEG (2, 5, and 7%) showed symptoms of hyperhydricity (a plant disorder in which shoots presents a rigid translucent aspect with little internodes). Usually, this disorder appears in in vitro cultures and is caused by different factors (e.g., water availability, growth regulators) [6]. This phenomenon can explain the highest biomass produced when cultures were submitted to drought stress. Similar signs of hyperhydricity were evidenced in *S. lerrifolia* cultures exposed to PEG stress [46].

Among the three levels of PEG tested, 7% induced the highest negative effects in *T. lotocephalus* cultures, therefore this concentration was selected to study the potential of three different agents (Fe_3_O_4_ NPs, SA, and MeJA) in alleviating drought stress. Nevertheless, as observed in Table 1, none of the agents tested were capable of reversing the negative impacts of PEG on the shoot’s length, with MeJA still potentiating the negative effects in this parameter (6.67 ± 0.47 mm). Similar results were obtained in *Oryza sativa* shoots stressed with 3% PEG 6000, in which MeJA (5 mM) reduced the length of the shoots. Nevertheless, the three agents tested reduced the signs of hyperhydricity of the shoots cultured in media with 7% PEG (Figure 1). Fe_3_O_4_ NPs, SA, and MeJA were also added to PEG-free culture media to investigate their effects in non-stressful conditions. Results indicated that Fe_3_O_4_ NPs improved shoot number and biomass production, in comparison to the control. It is known that iron has a great influence on the growth and development of plants, and its nanoscale application in in vitro cultures facilitates its absorption and plant nutritional balance [17,48,49]. Furthermore, the application of these NPs has been demonstrated to improve the uptake of important macronutrients in some plants [49,50]. The results showed that SA decreased 2.4-fold *T. lotocephalus* shoot fresh weight (FW). Distinct findings were obtained by Mozafari et al. [48] who compared the influence of SA (0.05 mM) and Fe_3_O_4_ NPs (0.08 ppm) on the biomass produced by in vitro cultures of strawberry. In that case, the authors demonstrated more beneficial effects of SA than Fe_3_O_4_ NPs, although they used a much lower concentration of iron NPs (0.08 mg/L) than that used in this study (10 mg/L). Using concentrations higher than those tested in this study, Karamian et al. [9] observed that 100 µM of SA significantly improved the biomass of *Verbascum sinuatum* L. shoots; however, 3 mM did not affect the growth of *Impatiens walleriana* L. [51]. Similar to SA, MeJA seems to exert an inhibitory effect on *T. lotocephalus* shoots’s length (11.7 ± 0.56 mm), in accordance with previous results observed in *V. sinuatum* [9]. Interestingly, enhanced shoot length was evidenced in *Glycyrrhiza glabra* cultures by MeJA (0.1–2 mM) after 24 h of exposure, but after 48 h it decreased significantly [52]. These distinct outcomes prove that the efficacy of the tested agents is affected by several factors, such as plant species, culture type, time of exposure, and concentration.

### 3.2. Physiological and Biochemical Traits

Photosynthesis is a crucial process for ideal plant development, metabolism, and biomass production, which depends directly on plant pigment levels. The obtained results showed that drought stress induced by PEG had damaging effects on photosynthetic pigment contents (Table 1). The highest contents were observed in the control, and the increasing drought stress progressively reduced total chlorophylls and carotenoid contents, with 7% PEG inducing a reduction of 51.35% and 56.25%, respectively. A decline in photosynthetic pigment levels under PEG stress was also observed in other species [4,46,47,51,53]. MeJA caused a reversion of the 7% PEG effects on the levels of total chlorophylls (from 0.38 ± 0.02 to 0.51 ± 0.03 mg/g_FW_). In contrast, in *V. sinuatum* cultures treated with PEG, the addition of 200 µM MeJA decreased the pigments’ contents [9]. The reduced amount of pigment under abiotic stress may be attributed to the high amount of ROS, which results in damage to the plant cells. ROS, which includes hydrogen peroxide (H_2_O_2_), hydroxyl radical (OH^−^), superoxide anions (O_2_^−^), and singlet oxygen (^1^O_2_) [54], act as signaling molecules to induce the expression of several genes and pathways [55].

As shown in Table 1, there were no significant differences between the H_2_O_2_ levels of the control and the media containing 2% and 5% PEG, but the H_2_O_2_ accumulation was significantly increased by 7% PEG (from 0.63 ± 0.00 to 1.28 ± 0.13 µmol/g_FW_). Likewise, cellular damage marked by the elevated levels of H_2_O_2_ was reported in several species under PEG-induced drought stress [46,47,56,57]. MeJA was able to reduce the high H_2_O_2_ accumulation induced by 7% PEG in *T. lotocephalus* shoots (from 1.28 ± 0.13 to 0.94 ± 0.11 µmol/g_FW_). Contrary to these results, MeJA negatively affected the redox status of *V. sinuatum*, increasing the content of H_2_O_2_ and being not able to alleviate the harmful consequences of drought stress [9]. The addition of Fe_3_O_4_ NPs and SA, to PEG-free and 7% PEG-containing media, also induced a significant accumulation of H_2_O_2_ in comparison with the control in *T. lotocephalus* shoots. In agreement with our results, it has been previously reported that SA significantly increases the levels of H_2_O_2_ [56]. A significantly high accumulation of H_2_O_2_ was reported in *Artemisia aucheri* treated with PEG 4% and 0.1 mM SA [56]. Moreover, SA treatments showed a positive effect in decreasing H_2_O_2_ contents in *I. walleriana* under 1–4% PEG [51]. Excessive oxidative stress was also observed in other species subjected to different metal oxide NPs [9,17,58,59], which denotes the ROS effects induced by metals binding with proteins presenting SH bonds, which are found in plants.

Under drought stress, to regulate cellular redox status due to excessive ROS production and cellular osmotic adjustment, as well as to stabilize membranes and proteins, plants accumulate several osmoprotectants, such as proline [54]. Thus, proline accumulation under drought conditions is an adequate marker of stress tolerance [53,60]. In this investigation, 5% and 7% PEG caused a significant rise in proline accumulation, compared to the control. The values ranged from 0.59 ± 0.01 µmol/g_FW_ in the control treatment to 4.55 ± 0.33 µmol/g_FW_ in the greatest percentage of PEG treatment (Table 1). Similar results were achieved in other species [46,47,56,57]. Interestingly, in *T. vulgaris* proline accumulation increased until 6% PEG but decreased significantly at 8%, probably due to the greater proline utilization outpacing their biosynthesis [4]. In *T. lotocephalus*, proline accumulation triggered by drought stress was accompanied by H_2_O_2_ accumulation in a dose-dependent manner, which demonstrates the protective role of proline against ROS production. This evidence is consistent with that observed in in vitro cultures of *A. orientalis* [47], *S. leriifolia* [46], and *I. walleriana* [51]. In higher plants, this osmoprotectant derives from glutamate, and its biosynthesis occurs in chloroplasts or cytosol [61]. Since the photosynthetic pigments’ degradation increases with increasing PEG concentration (which may be due to chloroplast damages), the majority of the proline biosynthesis in *T. lotocephalus* under drought stress probably occurs in the cytosol. Although Fe_3_O_4_ NPs, SA, and MeJA did not influence proline content per si, MeJA increased proline accumulation in PEG-stressed cultures (5.37 ± 0.25 µmol/g_FW_), suggesting the protective role of this signaling molecule in improving drought tolerance. Similar results were obtained in two cultivars of *O. sativa* seedlings under PEG stress, in which the priming with 2.5 and 5 mM MeJA significantly enhanced the proline accumulation [60].

### 3.3. Chemical Composition of the Shoots by Fourier-Transform Infrared (FTIR) Spectroscopy

**Figure 2:**
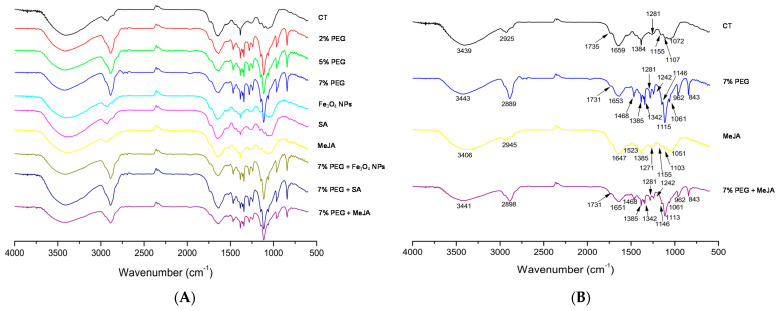
(**A**) FTIR spectra (600–4000 cm^−1^) of *Thymus lotocephalus* shoots cultured in media with 0 (control, CT), 2, 5, or 7% PEG, Fe_3_O_4_ nanoparticles (NPs), salicylic acid (SA), or methyl jasmonate (MeJA) and a combination of PEG (7%) with Fe_3_O_4_ NPs, SA and MeJA; (**B**) FTIR spectra of *T. lotocephalus* shoots cultured in media with 0 (control, CT), 7% PEG, MeJA or 7% PEG + MeJA with the identification of the bands (the corresponding bonds and functional groups are presented in Table 2).

FTIR is a powerful spectroscopic tool used to obtain detailed information about the major functional groups in the chemical composition of a sample. In plants, the initial responses to biotic or abiotic stresses can be assessed by changes in functional groups, which present characteristic frequencies in the infrared spectrum [62]. In this way, to investigate the molecular structural changes of the *T. lotocephalus* cells caused by PEG stress and the mitigating agents (Fe_3_O_4_ NPs, SA, and MeJA), FTIR analysis was performed. The FTIR spectra (4000 to 600 cm^−1^) are shown in Figure 2A,B and the wavenumbers of characteristic bands and corresponding functional groups are listed in Table 2.

FTIR spectra showed some chemical differences between the control and the shoots growing in all media containing PEG (with or without mitigating agents) (Figure 2A). PEG stress showed additional characteristic bands, namely at 1468, 1342, 1242, 1115, 962, and 843 cm^−1^ (Figure 2B), that are associated with phenyl groups or terpenoids (Table 2). According to Schulz and Baranska [35], apparently, drought stress affects positively the biosynthesis of some specific volatile compounds, namely 1,8-cineol (peak 843 cm^−1^), lutein (peak 962 cm^−1^), and especially citronellal (peak 1115 cm^−1^) compounds with characteristic bands that appeared in our samples. These results were also consistent with the results of Sevindik et al. [63] who observed that FTIR analysis of *Ocimum basilicum* (Lamiaceae) irrigated with PEG 6000 exhibited major alterations in the functional groups corresponding to bands below 1400 cm^−1^. FTIR spectra from shoots cultivated in the control medium and PEG-free media containing Fe_3_O_4_ NPs, SA, or MeJA were similar (Figure 2A,B), indicating that these agents do not induce appreciable chemical modifications evaluated with this technique. Similar results were obtained in *Rosmarinus officinalis*, another Lamiaceae species [34].

### 3.4. Phenolics Biosynthesis and Biological Activity of the Extracts

#### 3.4.1. Total Phenolic Contents by F-C Method

Many abiotic stresses, including drought induced by PEG, frequently affect the production of phenolic compounds as a reaction to oxidative injury [53,64,65]. In this work, all PEG concentrations significantly reduced the total phenolic content (TPC) in the extracts obtained by F-C method (Table 3). According to Moradi et al. [2], when sensitive plants are exposed to stress conditions they usually present a higher accumulation of metabolites than tolerant plants, which can evidence a certain drought tolerance of *T. lotocephalus* cultures. In accordance with these results, PEG also showed a negative impact on phenolics accumulation in *Taxus baccata* (1%, 2%, and 3% PEG) [57] and *V. sinuatum* (−0.6 MPa osmotic potential) [9]. However, in *T. vulgaris* grown in vitro [4] or ex vitro [12,55] conditions, drought significantly improved TPC compared to the control treatment. The exposure of stressed plants (7% PEG) to SA (53.8 ± 3.07 mg_GAE_/g_DW_) and MeJA (57.4 ± 2.34 mg_GAE_/g_DW_) caused a significant rise in TPC in comparison with the 7% PEG treatment (46.8 ± 0.42 mg_GAE_/g_DW_). Other authors also reported the beneficial effects of MeJA on the phenolic compound accumulation in plants under drought stress [9,66]. TPC significantly increased in non-stressed cultures treated with Fe_3_O_4_ NPs, SA, and MeJA. Similar findings were achieved by other authors [6,9,66], reinforcing the role of these agents as elicitors. MeJA was shown to be the best elicitor for *T. lotocephalus* shoot cultures, enhancing phenolics accumulation in 38.8%. The same was obtained in *Rubus idaeus* [67], *Mentha × piperita* [66], and *Brassica rapa* L. ssp. *chinensis* [68] treated with MeJA. Recently, Kianersi et al. [69] tested distinct concentrations of MeJA (10, 100, 150, and 200 µM) in different *Thymus* species (*T. vulgaris*, *T. migricus*, and *T. daenensis*) and observed that the maximum phenolics accumulation was achieved using 100 µM MeJA in those species.

#### 3.4.2. Phenolic Profile Analysis by HPLC-HRMS

Secondary metabolites are substances produced by plants that make them competitive in their environment and are essential in responding to biotic and abiotic stresses. Phenolic compounds are one of the main classes of secondary metabolites with important biological properties in plants [5]. In the present study, the phenolic profile of *T. lotocephalus* extracts obtained from shoots cultivated in media with PEG and/or with different mitigating agents, obtained for the first time using NADES, was analyzed by HPLC-HRMS. A total of twenty-six phenolics (19 phenolic acids, five flavonoids, a coumarin derivative, and a hydroxybenzaldehyde) were identified and quantified in *T. lotocephalus* extracts (Table 4 and Supplementary Tables S1 and S2).

To the best of our knowledge, this is the first time that epigallocatechin gallate, methyl 6-*O*-galloyl-β-d-glucopyranoside, theaflavic acid, methylrosmarinic acid (I and II), dihydromorelloflavone, protocatechuic aldehyde, melitric acid B and salviaflaside are identified in *Thymus* genera. Nevertheless, melitric acid B, methylrosmarinic acid, salviaflaside and protocatechuic aldehyde, were previously identified in other genera belonging to the Lamiaceae family, namely *Melissa* and *Salvia* [70,71,72]. Salvianolic acid F and sagerinic acid, although being identified for the first time in *T. lotocephalus*, were detected in other *Thymus* species (*T. zygis*, *T. pulegioides*, *T. fragrantissimus*, and *T. herba-barona*) [73]. As reported in previous studies with this species [6,15,17], rosmarinic acid was the major biocompound identified in the extracts (with a range of 12.5 g/kg in 7% PEG + MeJA to 58.2 g/kg in MeJA) (Table 4). Rosmarinic acid has been demonstrated to have important biological properties, such as antioxidant, anti-inflammatory, antitumor, neuroprotective, and antimicrobial [74,75]. This phenolic acid is utilized as a food and cosmetic ingredient and many pharmaceutical applications have also been reported [76]. The second most abundant compounds were epigallocatechin gallate (EGCG) (flavonoid) and methyl 6-*O*-galloyl-β-d-glucopyranoside (phenolic acid), although both eight times less abundant than rosmarinic acid in the control. Although the greatest total phenolic content (and rosmarinic acid) was achieved in the MeJA extract, it was not significantly different from the other agents (SA and Fe_3_O_4_ NPs) and control extracts, as observed when TPCs were determined by F-C method. Even though a high correlation (*p* < 0.01) between TPC by F-C assay and HPLC exists, there are some differences in the statistical analysis, which can be explained by the poor specificity of F-C reagent that can oxidize other substances, overestimating the results [77].

Regarding the effect of drought on phenolics biosynthesis, as noted for morphological and physiological traits and in agreement with F-C results, PEG significantly reduced the content of almost all phenolic compounds (especially rosmarinic acid), compared to the control. Likewise, rosmarinic acid content decreased by water stress in other Lamiaceae species, such as *O. basilicum* [78], *Melissa officinalis* [79,80], and *Salvia miltiorrhiza* [81]. On the other hand, the production of flavonoids (epigallocatechin gallate, dihydromorelloflavone, and theaflavic acid) was not affected by drought. It was observed that MeJA showed beneficial effects on phenolic acids production in stressed plants, improving the synthesis of methyl 6-O-galloyl-β-D-glucopyranoside (two-fold) and salvianolic acids (A and B). Under non-stress conditions, MeJA enhanced two-fold the biosynthesis of salvianolic acids, caffeic acid, and protocatechuic aldehyde. This agent also significantly improved the production of salvianolic and caffeic acids in *Salvia* species [82,83].

#### 3.4.3. Potential Properties of the Extracts for Skincare Products and Correlation with Phenolic Composition

##### Antioxidant Activity

**Figure 3:**
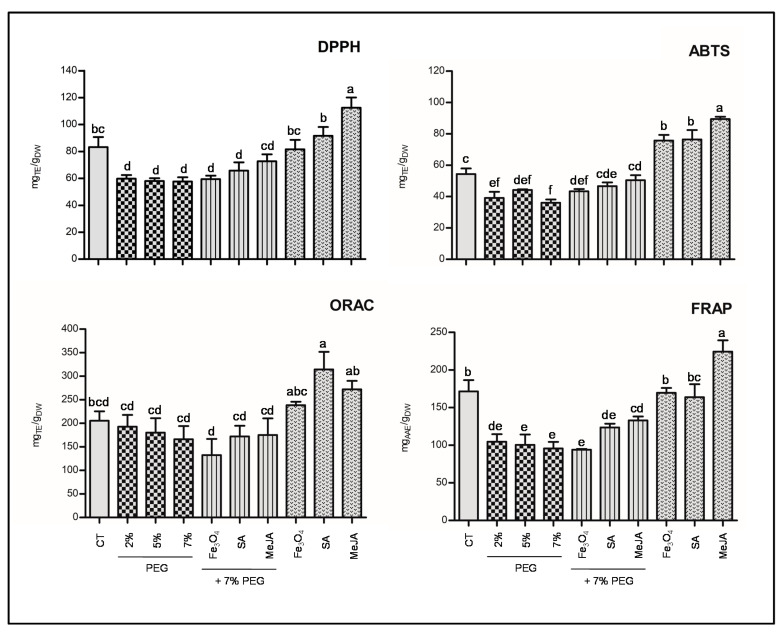
Antioxidant activity evaluated by 2,2-diphenyl-1- picrylhydrazyl (DPPH), 2,2-azino-bis(3-ethylbenzothiazoline-6-sulfonic acid) (ABTS), oxygen radical absorbance capacity (ORAC), and ferric reducing antioxidant power (FRAP) methods of the extracts from *T. lotocephalus* shoots cultured in media with 0 (control, CT), 2, 5, or 7% of PEG, 7% PEG + Fe_3_O_4_ nanoparticles (NPs), 7% PEG + salicylic acid (SA), or 7% PEG + methyl jasmonate (MeJA), Fe_3_O_4_ NPs, SA, or MeJA. Values are presented as mean ± SE. Different letters (a–f) in each graph bars specify significant differences (*p* < 0.05, Tukey’s new multiple test).

Since plant extracts are extraordinarily complex multicomponent mixtures, the antioxidant capacity of *T. lotocephalus* extract was evaluated using more than one assay (ORAC, FRAP, DPPH, and ABTS) (Figure 3). The ORAC measures the capacity of an antioxidant to break hydrophilic and lipophilic chains in the presence of peroxyl radicals. FRAP assay determines the capacity of the extracts to reduce ferric ion (Fe^3+^) to ferrous ion (Fe^2+^). Finally, DPPH and ABTS quantify the scavenging capability against stable free radicals.

**Figure 4:**
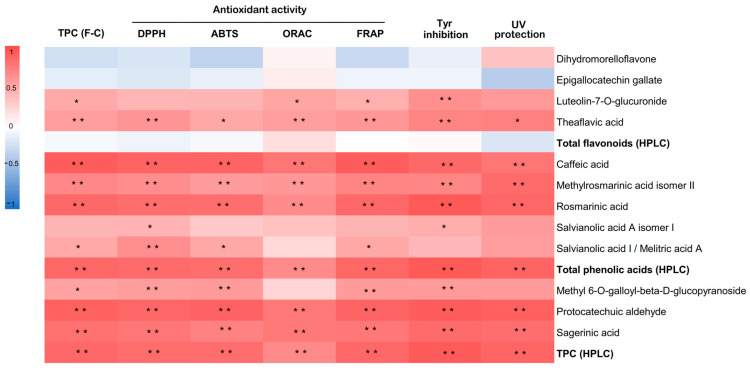
Heat map corresponding to Pearson’s correlation coefficients between antioxidant activity measured DPPH, ABTS, ORAC, and FRAP, tyrosinase (Tyr) inhibition capacity, UV protection ability, total phenolic contents measured by F-C and HPLC, total flavonoids (HPLC), total phenolic acids (HPLC) and individual phenolic compounds (HPLC). ** Correlation is significant (*p* < 0.01). * Correlation is significant (*p* < 0.05).

Similar to TPCs, in all assays, the antioxidant activity of the *T. lotocephalus* extracts were significantly reduced by all concentrations of PEG compared to the control, except in the ORAC assay, in which no significant differences were observed. These results confirm those previously observed for *T. vulgaris*, in which antioxidant activity accessed by FRAP decreased to half at 8% PEG [4]. Otherwise, an increase in antioxidant activity was reported in *S. rebaudiana* under rising concentrations of PEG (0.5, 1, 2, 4%) [64], as well as in *T. vulgaris* [12] and *Mentha piperita* [66] grown under water deficit. MeJA increased the antioxidant capacity of the cultures subjected to PEG stress in almost all the assays. Similar results were obtained in infusions prepared from peppermint plants grown under water deficit, in which MeJA (50 µM) revealed the greatest capability to scavenge ABTS^+^ and DPPH^•^ radicals [66]. Regarding the impact of the tested agents under non-stress conditions, similar to TPC by F-C assay, the antioxidant activity was significantly higher in MeJA treatment in all the assays, except in ORAC in which SA, Fe_3_O_4_ NPs and control demonstrated a similar capacity to quench free radicals by hydrogen donation. This exception can be justified by the distinct mechanisms implicated in the various methods. The increase in antioxidant activity was observed in extracts from other plants grown with MeJA [67,68]. The extracts obtained from the cultures treated with Fe_3_O_4_ NPs and SA showed higher antioxidant capacities (ABTS and ORAC) compared to the control. A similar trend was obtained in our previous works, in which Fe_3_O_4_ NPs [17] and SA [6] improved the antioxidant capability of *T. lotocephalus* methanolic extracts. Since this is the first study investigating the impact of MeJA, there are no reports in the literature to compare the obtained results. A strong correlation was established between all antioxidant results (DPPH, ABTS, FRAP, and ORAC) and TPC by HPLC (*p* < 0.01) (Figure 4) showing that phenolic compounds, especially phenolic acids, are the major contributors to *T. lotocephalus* antioxidant activities. The correlation between antioxidant activity and this class of bioactive compounds has been recently reported in *Thymus* species [6,17,84,85]. Although rosmarinic acid possesses a great significance to the antioxidant activity of the extracts because it is the major compound, the strong correlations (*p* < 0.01) between other minor compounds and antioxidant activity assays, such as sagerinic acid, protocatechuic aldehyde, and caffeic acid, equally demonstrate some influence of these compounds to this activity.

##### Extracts as Anti-Depigmentation Agents

The depigmentation or whitening effects of a product are related to its capacity to inhibit Tyr enzyme activity. Thus, the potential of *T. lotocephalus* extracts to inhibit this enzyme were evaluated in this study (Table 3). The Tyr inhibition activity varied from 5.98 ± 1.09 to 34.1 ± 0.31 mg_KAE_/g_DW_. Extracts from cultures exposed to PEG demonstrated the lowest Tyr inhibition. The three agents tested in this work under non-stressed conditions, particularly MeJA, significantly improved the anti-Tyr activity in *T. lotocephalus*. A strong correlation (*p* < 0.01) was observed between Tyr inhibition and the total phenolic compounds (HPLC) (Figure 4), which were similarly reported in other studies [17,18]. Rosmarinic acid was the phenolic compound that demonstrated the highest correlation with Tyr inhibition (r = 0.943). This phenolic acid was previously defined as a good inhibitor of this enzyme [86]. Although present in smaller amounts, protocatechuic aldehyde, caffeic acid, sagerinic acid, theaflavic acid, and methylrosmarinic acid strongly contributed (*p* < 0.01) to inhibiting Tyr. The inhibitory capacity of protocatechuic aldehyde and caffeic acid against Tyr was also previously reported by Ko and Lee [87] and Crespo et al. [88], respectively.

##### Extracts as Ultraviolet (UV) Protecting Agents

**Figure 5:**
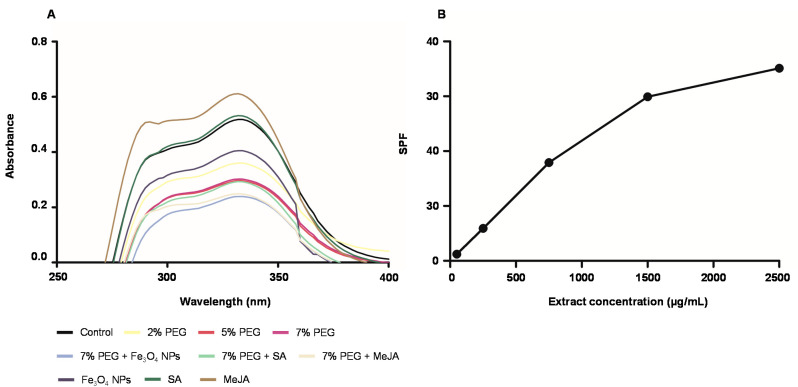
(**A**) UV-Vis absorption spectra of the extracts (250 μg/mL) from *T. lotocephalus* shoots cultured in media with 0 (control), 2, 5, or 7% of PEG, Fe_3_O_4_ nanoparticles (NPs), salicylic acid (SA), methyl jasmonate (MeJA), 7% PEG + Fe_3_O_4_ NPs, 7% PEG + SA, or 7% PEG + MeJA; (**B**) Sun Protection Factor (SPF) of the extract from *Thymus lotocephalus* shoots cultured in media with MeJA (50 µM).

In cosmetics, the capacity to absorb UV-A and UV-B radiation is an outstanding starting point to consider the extract as a natural sun protective agent [22]. To assess the potential of *T. lotocephalus* extracts as UV-protecting agents, different concentrations of the extracts were tested and their capacity to absorb UV-A (315–400 nm) and UV-B (200–280 nm) radiation was evaluated spectrophotometrically. The UV–vis spectra of the extracts at 250 μg/mL are shown in Figure 5A.

All *T. lotocephalus* extracts exhibited the capacity to absorb UV radiation, although the extract from cultures grown with MeJA showed the highest capacity with a band at the wavelength of 332 nm (Abs = 0.611). Even though the greatest ability of this extract is to absorb UV-A rays (55.79%), it also demonstrated a good capacity to inhibit UV-B radiation (36.86%). Similar to antioxidant and anti-melanogenic activities results, all cultures exposed to PEG demonstrated the worst photoprotective capacity. A strong correlation was found between several phenolic compounds and UV protecting activity (*p* < 0.01) (Figure 4), such as protocatechuic aldehyde, and rosmarinic, methylrosmarinic, sagerinic, and caffeic acids. Rosmarinic and caffeic acids were previously reported as photoprotective agents against UV and other ionizing radiations [89,90].

Since MeJA extract showed the highest ability to absorb UV radiation, the sun protection factor (SPF) was only calculated for this extract at different concentrations (50, 250, 750, 1500, and 2500 µg/mL). Compared to the in vivo models for the determination of SPF, the in vitro models present several advantages since they are simple, reproducible, fast, and avoid subject UV exposure [91]. The UV radiation in the range of 290–320 nm retains the highest biological activity to induce skin damage, such as burning, photoaging, and cancer, so the SPF of a product is evaluated in these wavelengths. According to Yakoubi et al. [92] the rating of sun-protective activity of sunscreens can be determined according to SPF values in minimum (2–12), moderate (12–30), and high (≥30). The SPF values of the *T. lotocephalus* extract increased in a concentration-dependent manner (Figure 5B). Fifty µg/mL (SPF = 1.20) and 250 µg/mL (SPF = 5.93) of *T. lotocephalus* extract resulted in minimum, 750 µg/mL (SPF = 17.92) in moderate, and finally, 1500 µg/mL (SPF = 29.92) and 2500 µg/mL (SPF = 35.13) in high photoprotective activities. Other plant extracts have been reported in the literature as good agents to absorb UV radiation, such as ethanolic and aqueous-glycerin extracts of *Plantago lanceolata* [22] and polyol extracts of *Camellia oleifera* [93]. With regards to *Thymus* extracts, as far as is known, there are no previous reports describing their capacity to absorb UV radiation and its SPF evaluation. Although NADES are considered green solvents, only a few can be applied in cosmetics because of safety or regulatory reasons, especially in Europe [25]. This is the case of the mixture used in the present study (proline: lactic acid, 1:1), which is accepted by the European Cosmetic Regulation EC No.1223/2009 to be used in cosmetic products formulations.

#### 3.4.4. Multivariate Analyses: Cluster Analysis and Principal Component Analysis (PCA)

**Figure 6:**
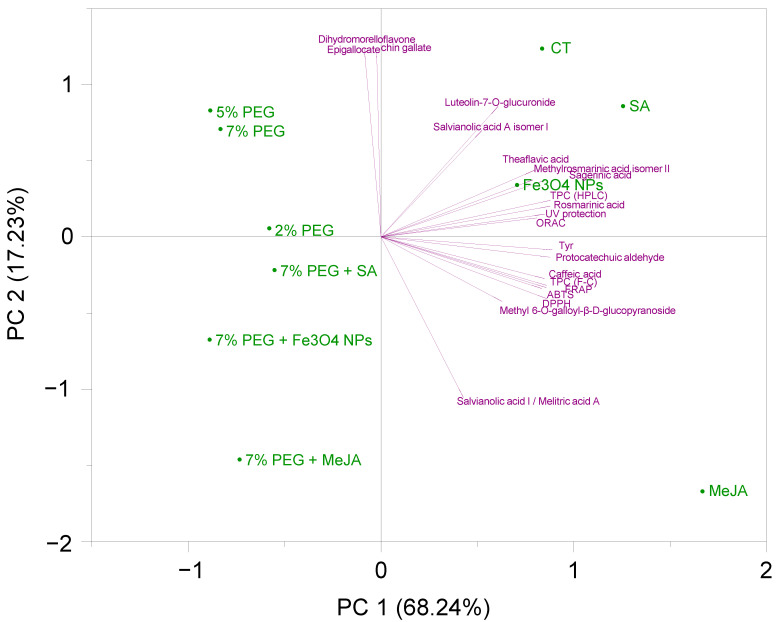
Principal component analysis (PCA) biplot of the different polyphenols and bioactivities studied in extracts from *T. lotocephalus* shoots cultured in media with 0 (control, CT), 2, 5, or 7% of PEG, Fe_3_O_4_ nanoparticles (NPs), salicylic acid (SA), methyl jasmonate (MeJA), 7% PEG + Fe_3_O_4_ NPs, 7% PEG + SA, or 7% PEG + MeJA.

Principal Component Analysis (PCA) is used to decrease the dimensionality of a multivariate data set in a few principal components (PC), which summarize the predominant patterns in the data. PCA was based on the individual and total phenolic content (F-C and HPLC) and the biological properties (photoprotective, anti-melanogenic, and antioxidant) of the extracts. The analysis can be observed in Figure 6 in a PCA biplot [PCA score plot (green) + loading plot (purple)] form. Each point on the score plot characterizes the different extracts, and each point on the loading plot indicates the contribution of each variable to the score. The first two PC described 85.47% of the total variation in the dataset, explaining the first principal component (PC1) 68.24% of the data variability, and the second principal component (PC2) 17.23%. The score plot of the first two principal components demonstrated a clear grouping of the extracts by PEG influence. It is possible to observe a clear separation between samples grown with PEG (negative PC1 values, second and third quadrants) and samples grown without PEG (positive PC1 values, first and fourth quadrants). Similarly, PCA analysis of *S. rebaudiana* in vitro plants subjected to PEG 6000 (2.5, 5, 7, and 10%) revealed a pronounced separation between the controls and the treatment groups [94].The results indicate that the different agents (Fe_3_O_4_ NPs, SA, and MeJA) and PEG concentrations (2, 5, and 7%) added to the culture media present a different ability to induce the production of distinct classes of phenolics. In general, the major number of phenolic compounds and consequent biological properties were produced in higher amounts in the media containing the different mitigating agents (Fe_3_O_4_ NPs, SA, and MeJA), or in the control. Positioned at higher positive values of PC1, MeJA, SA, and PC2, control presented the highest contents in rosmarinic acid and its derivatives methyl rosmarinic (isomer II) and sagerinic acids. The control extract stands out for its content in the flavonoid luteolin-7-O-glucuronide and the rosmarinic derivative salvianolic acid A (isomer I), while SA and MeJA showed the highest contents in protocatechuic aldehyde and caffeic acid. Furthermore, the MeJA sample, situated in the fourth quadrant and with the greatest influence on PC1 and PC2, presented the highest difference among samples, and was shown to be the best agent to promote the production of salvianolic acid I/melitric acid A, exhibiting the highest abilities as UV protection, Tyr inhibition and with the highest TPC (F-C method) and antioxidant capacity by FRAP, DPPH, and ABTS.

**Figure 7:**
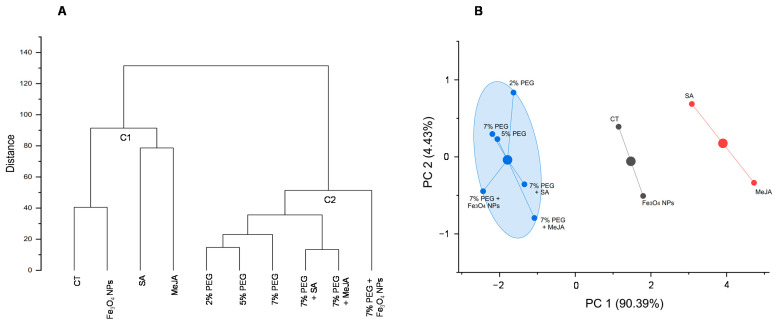
(**A**) Hierarchical cluster analysis based on the biological properties (photoprotective, anti-melanogenic, and antioxidant activities) of the extracts from *T. lotocephalus* shoots cultured in media with 0 (control, CT), 2, 5, or 7% of PEG, Fe_3_O_4_ nanoparticles (NPs), salicylic acid (SA), methyl jasmonate (MeJA), 7% PEG + Fe_3_O_4_ NPs, 7% PEG + SA, or 7% PEG + MeJA; (**B**) K-means cluster analysis according to the biological properties (photoprotective, anti-melanogenic, and antioxidant activities) of the *T. lotocephalus* extracts.

Hierarchical cluster analysis and K-means cluster analysis are two multivariate analyses to recognize the clustering pattern and group objects according to the similarities among samples. A cluster analysis from data of total phenolic content (F-C) and biological properties (photoprotective, anti-melanogenic, and antioxidant) of the extracts provided good separation among the samples (Figure 7A,B). The dendrogram (Figure 7A) grouped the samples into two principal clusters: cluster 1 (C1) included control and Fe_3_O_4_ NPs, SA, and MeJA, and cluster 2 (C2) comprised all PEG-treated samples (2, 5, 7%, and 7% PEG + agents). Both hierarchical (Figure 7A) and K-means (Figure 7B) clusters showed close relationships in the two sub-groups of the first cluster, namely between the control and Fe_3_O_4_ NPs, and between the signaling molecules SA and MeJA. Regarding PEG treatments, both analyses grouped all samples from PEG media (with or without agents) into one cluster, showing the strong role of this osmotic agent in inducing drought stress in *T. lotocephalus* shoot cultures, and its huge influence on the biological activities of the extracts. Moreover, it suggests that the agents tested in this study and/or their concentrations were not efficient enough to mitigate the stress produced in the plant by the use of PEG.

## 4. Conclusions

**Figure 8:**
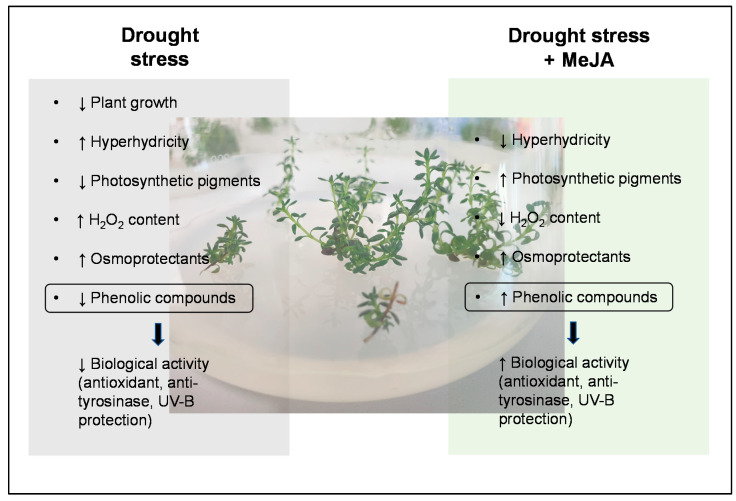
The overall response of *Thymus lotocephalus* in vitro cultures to drought stress induced by PEG and the positive effects of methyl jasmonate (MeJA) in relieving damage caused by stress.

The results obtained suggest that PEG added to culture media has a considerable impact on *T. lotocephalus* in vitro cultures, namely increasing the oxidative stress and causing direct damage on shoots growth, photosynthetic pigments, and bioactive compounds synthesis and bioactivity (Figure 8). Among the different agents tested, the addition of MeJA to the culture media played a prominent role in the mitigation/reduction of some drought harm (Figure 8). MeJA was also shown to be useful as an elicitor, improving the biosynthesis of some phenolic compounds and consequent biological properties of the extracts. Moreover, this study showed that *T. lotocephalus* extracts prepared with Natural Deep Eutectic Solvents (NADES) have the potential to be used in cosmetics due to their ability to absorb UV radiation, as well as antioxidant and depigmentation properties. Nevertheless, more detailed parameters must be carefully investigated, such as concentration, stability, compatibility, and toxicity of the extracts. Overall, although our findings indicate that in vitro culture proves to be an adequate tool for a first estimation of the effect of drought in this species, it will be important, in future works, to evaluate the response of plants to drought stress under ex vitro conditions, and to understand if PEG stress indeed alters the production of terpenoids, as FTIR studies suggest.

## Figures and Tables

**Table 1:** Shoot growth and total chlorophyll (Cltotal), carotenoids (Crt), hydrogen peroxide (H_2_O_2_), and proline contents of *Thymus lotocephalus* López and Morales shoots cultured in media with 0 (control), 2, 5, or 7% of PEG, 7% PEG + Fe_3_O_4_ nanoparticles (NPs), 7% PEG+ salicylic acid (SA), or 7% PEG+ methyl jasmonate (MeJA), Fe_3_O_4_ NPs, SA, or MeJA.

Treatment	No. Shoots	Length of the Longest Shoot (mm)	Fresh Weight (g)	Dry Weight (mg)	Cltotal (mg/gFW)	Crt (mg/gFW)	H_2_O_2_ (µmol/g_FW_)	Proline (µmol/g_FW_)
Control	7.94 ± 1.70 ^c^	48.3 ± 2.68 ^a^	1.40 ± 0.383 ^cd^	144 ± 26.6 ^c^	0.74 ± 0.02 ^a^	0.16 ± 0.01 ^ab^	0.63 ± 0.00 ^de^	0.59 ± 0.01 ^d^
PEG								
2%	33.2 ± 1.77 ^a^	22.9 ± 0.89 ^c^	3.69 ± 0.20 ^a^	417 ± 13.9 ^a^	0.59 ± 0.03 ^bc^	0.14 ± 0.00 ^abc^	0.43 ± 0.03 ^e^	1.39 ± 0.14 ^bcd^
5%	21.9 ± 1.72 ^b^	15.7 ± 0.80 ^d^	2.06 ± 0.11 ^bc^	295 ± 17.1 ^b^	0.50 ± 0.01 ^cdef^	0.12 ± 0.01 ^bcd^	0.79 ± 0.07 ^cde^	1.53 ± 0.16 ^bc^
7%	18.4 ± 2.32 ^b^	11.5 ± 0.43 ^de^	1.33 ± 0.10 ^cd^	318 ± 33.5 ^ab^	0.38 ± 0.02 ^f^	0.09 ± 0.01 ^d^	1.28 ± 0.13 ^bc^	4.55 ± 0.33 ^a^
7% PEG								
Fe_3_O_4_ NPs	4.71 ± 0.47 ^c^	10.3 ± 0.39 ^de^	0.59 ± 0.12 ^de^	93.3 ± 18.2 ^c^	0.45 ± 0.04 ^def^	0.11 ± 0.01 ^cd^	1.47 ± 0.08 ^b^	1.57 ± 0.13 ^bc^
SA	4.32 ± 0.93 ^c^	10.4 ± 0.95 ^de^	0.66 ± 0.14 ^de^	102 ± 13.4 ^c^	0.41 ± 0.02 ^ef^	0.10 ± 0.00 ^cd^	1.37 ± 0.00 ^b^	2.27 ± 0.25 ^b^
MeJA	3.44 ± 0.67 ^c^	6.67 ± 0.47 ^e^	0.21 ± 0.08 ^e^	49.1 ± 14.6 ^c^	0.51 ± 0.03 ^cde^	0.12 ± 0.00 ^cd^	0.94 ± 0.11 ^bcde^	5.37 ± 0.25 ^a^
PEG-free								
Fe_3_O_4_ NPs	25.4 ± 2.34 ^ab^	29.6 ± 1.10 ^b^	3.10 ± 0.35 ^ab^	320 ± 23.8 ^ab^	0.68 ± 0.01 ^ab^	0.17 ± 0.01 ^a^	1.16 ± 0.12 ^bcd^	0.76 ± 0.10 ^cd^
SA	8.11 ± 1.34 ^c^	13.8 ± 0.95 ^d^	0.59 ± 0.15 ^de^	89.0 ± 14.3 ^c^	0.57 ± 0.01 ^bcd^	0.17 ± 0.01 ^a^	2.45 ± 0.17 ^a^	1.12 ± 0.07 ^cd^
MeJA	7.83 ± 0.79 ^c^	11.7 ± 0.56 ^de^	1.12 ± 0.05 ^cde^	106 ± 2.70 ^c^	0.43 ± 0.04 ^ef^	0.10 ± 0.01 ^cd^	0.60 ± 0.10 ^e^	0.77 ± 0.06 ^cd^

Values are expressed as mean ± SE. For each variable, the values followed by different letters (a–f) are significantly different at *p* < 0.05 (Tukey’s New Multiple Range Test).

**Table 2:** FTIR spectral bands and functional groups of *Thymus lotocephalus* shoots cultured in media with 0 (control) or 7% PEG, methyl jasmonate (MeJA), or 7% PEG + MeJA reported in Figure 2B.

Wavenumber Range		Bond	Functional Group	References
Present Study (cm^−1^)	Reference (cm^−1^)			
3443–3406	3415–3369	O–H stretching	Alcohols	[32,33]
2945–2889	2920–2800	C–H stretching	Lipids, carbohydrates	[32,33,34,36]
1735–1647	1723–1607	C=O stretching, C–N stretching, COO– antisymmetric stretching	Proteins, lipids, carbohydrates	[32,34,35,36]
1468–1342	1454–1366	C–O stretching, C–C stretching, COO symmetric stretching, CH_2_ bending	Phenyl groups of aromatic compounds	[33,34,36]
1281–1271	1300–1260	C–O stretching	Hydroxyflavonoids	[34,36]
1242–1155	1270–1150	C–O stretching, C–N stretching	Acid or ester	[33,34]
1115–1051	1170–950	C–O and C–C stretching	Carbohydrates, flavonoids	[34,35,36]
962–843	980–960	C=H bending, C–H out-of-plane bending	Terpenoids	[33,34,35]

**Table 3:** Total phenolic content (TPC) and tyrosinase inhibitory capacity of extracts from *Thymus lotocephalus* shoots cultured in media with 0 (control), 2, 5, or 7% of PEG, 7% PEG + Fe_3_O_4_ nanoparticles (NPs), 7% PEG+ salicylic acid (SA), or 7% PEG + methyl jasmonate (MeJA), Fe_3_O_4_ NPs, SA, or MeJA.

Treatment	TPC (mg_GAE_/g_DW_)	Tyrosinase Inhibition (mg_KAE_/g_DW_)
**Control**	62.2 ± 1.19 ^cd^	21.72 ± 1.25 ^b^
PEG		
2% PEG	45.1 ± 2.61 ^ef^	9.59 ± 0.10 ^c^
5% PEG	45.0 ± 3.39 ^ef^	5.98 ± 1.09 ^c^
7% PEG	46.8 ± 0.42 ^ef^	9.03 ± 1.61 ^c^
**7% PEG**		
Fe_3_O_4_ NPs	43.4 ± 2.35 ^f^	10.8 ± 1.89 ^c^
SA	53.8 ± 3.07 ^de^	10.2 ± 0.60 ^c^
MeJA	57.4 ± 2.34 ^d^	8.46 ± 1.50 ^c^
**PEG-free**		
Fe_3_O_4_ NPs	68.8 ± 0.70 ^bc^	28.2 ± 1.27 ^ab^
SA	76.6 ± 0.32 ^ab^	29.2 ± 0.48 ^a^
MeJA	86.4 ± 1.09 ^a^	34.2 ± 0.31 ^a^

Values are expressed as mean ± standard error. In each column values followed by different letters (a–f) are significantly different at *p* < 0.05 (Tukey’s New Multiple Range Test).

**Table 4:** Qualitative and quantitative [mg/kg or g/kg (marked with *) of extract, mean ± SE] analysis by HPLC-HRMS of the phenolic profile from *Thymus lotocephalus* shoots cultured in media with 0 (control), 2, 5, or 7% of PEG, 7% PEG + Fe_3_O_4_ nanoparticles (NPs), 7% PEG + salicylic acid (SA), or 7% PEG + methyl jasmonate (MeJA), Fe_3_O_4_ NPs, SA, or MeJA.

Compound	Treatment									
	Control	PEG			7% PEG			PEG-Free		
		2%	5%	7%	Fe_3_O_4_ NPs	SA	MeJA	Fe_3_O_4_ NPs	SA	MeJA
**Phenolic acids**										
Salvianolic acid A isomer I	468 ± 1 ^a^	137 ± 6 ^bc^	146 ± 6 ^bc^	150 ± 8 ^bc^	107 ± 16 ^c^	288 ± 38 ^abc^	172 ± 10 ^bc^	339 ± 20 ^ab^	323 ± 103 ^ab^	172 ± 41 ^bc^
Salvianolic acid A isomer II	<LOQ	<LOQ	<LOQ	<LOQ	<LOQ	<LOQ	133 ± 6	<LOQ	<LOQ	227 ± 64
Salvianolic acid A isomer IV	<LOQ	<LOQ	<LOQ	<LOQ	<LOQ	<LOQ	<LOQ	<LOQ	<LOQ	149 ± 28
Salvianolic acid B/Salvianolic acid L isomer I	<LOD	<LOD	<LOD	<LOD	n.d.	<LOD	<LOD	<LOD	<LOD	<LOD
Salvianolic acid B/Salvianolic acid L isomer II	<LOQ	<LOQ	<LOQ	<LOQ	<LOQ	<LOQ	<LOQ	<LOQ	<LOQ	<LOQ
Salvianolic acid B/Salvianolic acid L isomer III	<LOQ	<LOQ	<LOQ	<LOQ	<LOQ	174 ± 8	127 ± 10	<LOQ	<LOQ	<LOQ
Salvianolic acid B/Salvianolic acid L isomer IV	<LOQ	<LOQ	<LOQ	<LOQ	<LOQ	<LOQ	126 ± 10 ^b^	<LOQ	180 ± 39 ^b^	391 ± 46 ^a^
Salvianolic acid C	<LOQ	<LOQ	<LOQ	<LOQ	<LOQ	<LOQ	<LOQ	<LOQ	143 ± 8	199 ± 62
Salvianolic acid F isomer I	<LOQ	n.d.	n.d.	n.d.	n.d.	<LOQ	n.d.	<LOQ	<LOQ	<LOQ
Salvianolic acid F isomer II	173 ± 23 ^a^	113 ± 20 ^a^	<LOQ	<LOQ	<LOQ	<LOQ	<LOQ	118 ± 20 ^a^	199 ± 2 ^a^	204 ± 3 ^a^
Salvianolic acid I/Melitric acid A	252 ± 8 ^b^	224 ± 12 ^b^	176 ± 9 ^b^	178 ± 12 ^b^	295 ± 17 ^ab^	199 ± 31 ^b^	307 ± 7 ^ab^	178 ± 6 ^b^	192 ± 56 ^b^	591 ± 158 ^a^
Total salvianolic acids *	0.89 ± 0.03 ^b^	0.47 ± 0.00 ^b^	0.32 ± 0.02 ^b^	0.33 ± 0.02 ^b^	0.40 ± 0.03 ^b^	0.66 ± 0.06 ^b^	0.86 ± 0.02 ^b^	0.64 ± 0.05 ^b^	1.04 ± 0.21 ^b^	1.93 ± 0.40 ^a^
Caffeic acid	46 ± 16 ^cd^	30 ± 8 ^cd^	20 ± 3 ^cd^	23 ± 3 ^cd^	8 ± 2 ^d^	35 ± 4 ^cd^	30 ± 1 ^cd^	54 ± 7 ^bc^	90 ± 3 ^ab^	112 ± 10 ^a^
Melitric acid B	<LOD	<LOD	<LOD	<LOD	<LOD	<LOD	<LOD	<LOD	<LOD	<LOD
Methyl 6-O-galloyl-β-D-glucopyranoside *	6.00 ± 1.25 ^a^	4.71 ± 0.56 ^a^	3.61 ± 0.19 ^a^	2.46 ± 0.24 ^a^	4.30 ± 0.43 ^a^	4.20 ± 0.30 ^a^	5.01 ± 0.08 ^a^	6.02 ± 0.26 ^a^	4.39 ± 1.29 ^a^	6.15 ± 1.11 ^a^
Methylrosmarinic acid isomer I	94 ± 4 ^a^	<LOQ	<LOQ	83 ± 2 ^a^	<LOQ	<LOQ	<LOQ	84 ± 4 ^a^	75 ± 18 ^a^	78 ± 7 ^a^
Methylrosmarinic acid isomer II	651 ± 19 ^a^	342 ± 30 ^ab^	272 ± 38 ^b^	420 ± 0 ^ab^	265 ± 16 ^b^	384 ± 11 ^ab^	204 ± 38 ^b^	394 ± 91 ^ab^	616 ± 150 ^a^	632 ± 38 ^a^
Rosmarinic acid *	53.0 ± 3.57 ^a^	16.8 ± 1.25 ^bc^	15.9 ± 1.04 ^bc^	23.9 ± 0.55 ^b^	23.5 ± 0.78 ^b^	20.3 ± 1.98 ^bc^	12.5 ± 0.16 ^c^	48.2 ± 1.95 ^a^	54.9 ± 2.05 ^a^	58.2 ± 2.36 ^a^
Sagerinic acid *	2.88 ± 0.03 ^ab^	0.85 ± 0.03 ^c^	0.72 ± 0.01 ^c^	0.97 ± 0.05 ^c^	0.63 ± 0.17 ^c^	0.84 ± 0.08 ^c^	0.55 ± 0.04 ^c^	1.98 ± 0.12 ^bc^	3.70 ± 0.70 ^a^	2.73 ± 0.38 ^ab^
Salviaflaside	<LOQ	<LOQ	<LOQ	<LOQ	<LOQ	<LOQ	<LOQ	<LOQ	<LOQ	<LOQ
Total phenolic acids *	63.5 ± 2.36 ^ab^	23.3 ± 0.75 ^cd^	20.8 ± 0.85 ^cd^	28.2 ± 0.87 ^cd^	29.1 ± 1.43 ^c^	26.4 ± 2.12 ^cd^	19.2 ± 0.26 ^d^	57.4 ± 2.28 ^b^	64.8 ± 2.59 ^ab^	69.8 ± 1.30 ^a^
**Flavonoids**										
Dihydromorelloflavone	218 ± 34 ^a^	216 ± 18 ^a^	231 ± 19 ^a^	217 ± 7 ^a^	183 ± 4 ^a^	190 ± 4 ^a^	157 ± 9 ^a^	194 ± 6 ^a^	208 ± 34 ^a^	175 ± 9 ^a^
Epigallocatechin gallate *	6.60 ± 1.69 ^a^	4.51 ± 0.29 ^a^	7.59 ± 0.95 ^a^	6.60 ± 0.70 ^a^	4.22 ± 0.57 ^a^	4.56 ± 1.23 ^a^	4.15 ± 0.00 ^a^	5.65 ± 0.26 ^a^	6.13 ± 1.50 ^a^	3.96 ± 0.35 ^a^
Luteolin	12 ± 0 ^a^	<LOQ	<LOQ	<LOQ	12 ± 0 ^a^	<LOQ	<LOD	<LOQ	13 ± 1 ^a^	<LOQ
Luteolin-7-O-glucuronide	157 ± 6 ^a^	68 ± 3 ^b^	44 ± 2 ^b^	48 ± 3 ^b^	39 ± 6 ^b^	48 ± 4 ^b^	20 ± 3 ^b^	156 ± 23 ^a^	154 ± 16 ^a^	52 ± 0 ^b^
Theaflavic acid *	1.22 ± 0.00 ^a^	1.06 ± 0.02 ^a^	0.956 ± 0.07 ^a^	0.94 ± 0.03 ^a^	0.97 ± 0.00 ^a^	0.93 ± 0.00 ^a^	0.78 ± 0.00 ^a^	1.15 ± 0.09 ^a^	1.20 ± 0.26 ^a^	1.21 ± 0.12 ^a^
Total flavonoids *	8.21 ± 1.64 ^a^	5.85 ± 0.28 ^a^	8.82 ± 0.86 ^a^	7.80 ± 0.71 ^a^	5.42 ± 0.60 ^a^	5.73 ± 1.22 ^a^	5.11 ± 0.00 ^a^	7.14 ± 0.37 ^a^	7.70 ± 1.19 ^a^	5.40 ± 0.24 ^a^
**Coumarin derivative**										
Herniarin	<LOD	<LOD	<LOD	<LOD	<LOD	<LOD	<LOD	<LOD	<LOD	<LOD
**Hydroxybenzaldehyde**										
Protocatechuic aldehyde	66 ± 6 ^bcd^	49 ± 4 ^cd^	29 ± 5 ^d^	23 ± 3 ^d^	21 ± 0 ^d^	32 ± 3 ^d^	25 ± 13 ^d^	82 ± 16 ^bc^	112 ± 17 ^ab^	135 ± 0 ^a^
Total phenolic compounds *	71.8 ± 4.00 ^a^	29.2 ± 0.47 ^bc^	29.6 ± 0.00 ^bc^	36.0 ± 1.57 ^b^	34.6 ± 0.86 ^bc^	32.1 ± 0.91 ^bc^	24.3 ± 0.28 ^c^	64.6 ± 2.26 ^a^	72.6 ± 3.80 ^a^	75.3 ± 1.06 ^a^

Notes: n.d.—not detected; LOD—limit of detection; LOQ—limit of quantification. The results were analyzed using a one-way analysis of variance (ANOVA) followed by Tukey’s New Multiple Range Test. Different letters (a–d) in each row and for each phenolic compound mean significant differences (*p* < 0.05) among extracts.

## Data Availability

The data presented are included within the article and in the Supplementary Materials.

## Supplementary Materials

The following are available online at https://www.mdpi.com/article/10.3390/antiox11081475/s1, Table S1: HPLC-HRMS data of identified phenolics in *Thymus lotocephalus* López and Morales extracts, Table S2: Summary of HPLC-HRMS criterion for quantification of phenolics in *Thymus lotocephalus* López and Morales extracts, Table S3: EE(λ) × I(λ) constants used in the determination of SPF for each wavelength [45].
