# Supplementary material for: Response of Thymus lotocephalus In Vitro Cultures to Drought Stress and Role of Green Extracts in Cosmetics
Source: Antioxidants (Basel). 2022 Jul 28;11(8):1475. doi: 10.3390/antiox11081475 (PMC9404771; doi:10.3390/antiox11081475)
Supplement: Supplementary file 1 [file antioxidants-11-01475-s001.zip › antioxidants-1821846-supplementary.pdf]

Response of *Thymus lotocephalus* in vitro cultures to drought stress and role of green extracts in cosmetics

Inês Mansinhos[1], Sandra Gonçalves[1], Raquel Rodríguez-Solana[1,2], Hugo Duarte[1], José Luis Ordóñez-Díaz[2], José Manuel Moreno-Rojas[2] and Anabela Romano[1,*]

[1]*MED – Mediterranean Institute for Agriculture, Environment and Development & CHANGE – Global Change and Sustainability Institute, Faculdade de Ciências e Tecnologia, Universidade do Algarve, Campus de Gambelas, 8005-139 Faro, Portugal*

[2] *Department of Food Science and Health, Andalusian Institute of Agricultural and Fisheries Research and Training (IFAPA), Avenida Menendez-Pidal, SN, 14004 Córdoba, Spain*

** Correspondence: aromano@ualg.pt; Tel.: +351289800910*

Supplementary information

Table S1. HPLC-HRMS data of identified phenolics in *Thymus lotocephalus* López and Morales extracts.

Peak	Compound identity	Chemical formula	Theoretical exact mass $[M-H]^-$ *(m/z)*	Delta ppm (error)	RT (min)	MSI MI level*
1	Methyl 6-O-galloyl-β-D-glucopyranoside [a, 1]	$C_{14}H_{18}O_{10}$	345.0793	-5.81	2.64	2
2	Protocatechuic aldehyde [d, 2]	$C_7H_6O_3$	137.0239	0.07	3.96	2
3	Caffeic acid [a]	$C_9H_8O_4$	179.0338	-0.14	4.81	1
4	Epigallocatechin gallate [b]	$C_{22}H_{18}O_{11}$	457.0765	0.27	5.34	1
5	Salvianolic acid C [a, 3]	$C_{18}H_{17}O_9$	377.0873	-0.74	5.37	2
6	Dihydromorelloflavone [b, 4]	$C_{30}H_{22}O_{11}$	557.1083	0.13	5.37	2
7	Salviaflaside [a, 3]	$C_{24}H_{26}O_{13}$	521.1288	0.12	8.1	2
8	Luteolin-7-O-glucuronide [b, 5]	$C_{21}H_{18}O_{12}$	461.0714	-0.03	8.67	2
9	Salvianolic acid B /Salvianolic acid L isomer I [a, 3]	$C_{36}H_{30}O_{16}$	717.1456	-2.62	9.22	2
10	Rosmarinic acid [a]	$C_{18}H_{16}O_8$	359.0761	0.77	10	1
11	Theaflavic acid [b, 6]	$C_{21}H_{16}O_{10}$	427.0636	-5.28	10.04	2
12	Sagerinic acid [a, 3]	$C_{36}H_{32}O_{16}$	719.1612	-1.21	10.07	2
13	Salvianolic acid A isomer I [a, 3]	$C_{26}H_{22}O_{10}$	493.1129	-0.76	10.6	2
14	Salvianolic acid A isomer II [a, 3]	$C_{26}H_{22}O_{10}$	493.1129	0.36	11.47	2
15	Melitric acid A / Salvianolic acid I [a, 3]	$C_{27}H_{22}O_{12}$	537.1034	-0.73	11.55	2
16	Salvianolic acid B /Salvianolic acid L isomer II [a, 3]	$C_{36}H_{30}O_{16}$	717.1456	-0.58	11.63	2
17	Herniarin [c, 7]	$C_{10}H_8O_3$	175.0389	-1.83	12.51	2
18	Methylrosmarinic acid isomer I [a, 3]	$C_{19}H_{18}O_8$	373.0921	1.14	12.56	2
19	Salvianolic acid A isomer IV [a, 3]	$C_{26}H_{22}O_{10}$	493.1129	-0.82	13.1	2
20	Salvianolic acid B /Salvianolic acid L isomer III [a, 3]	$C_{36}H_{30}O_{16}$	717.1456	-0.59	13.16	2
21	Luteolin [b]	$C_{15}H_{10}O_6$	285.0393	0.55	13.97	1
22	Methylrosmarinic acid isomer III [a, 3]	$C_{19}H_{18}O_8$	373.0921	0.98	13.97	2
23	Melitric acid B [a, 3]	$C_{27}H_{20}O_{11}$	519.0922	-0.63	14.3	2
24	Salvianolic acid B / Salvianolic acid L isomer IV [a, 3]	$C_{36}H_{30}O_{16}$	717.1456	-0.50	14.34	2
25	Salvianolic acid F isomer I [a, 3]	$C_{17}H_{13}O_6$	313.0712	1.61	14.6	2
26	Salvianolic acid F isomer II [a, 3]	$C_{17}H_{13}O_6$	313.0712	0.15	15.26	2

*Metabolite standards initiative metabolite identification (MSI MI) levels. Reference standards were available for all compounds identified at MSI MI level 1. Class of compounds: [a]Phenolic acids; [b]Flavonoids; [c]Coumarin derivative; [d]Hydroxybenzaldehydes. Results expressed as equivalents of gallic acid (1), protocatechuic acid (2), rosmarinic acid (3), quercetin (4), luteolin (5), catechin (6), *p* -coumaric acid (7).

Table S2. Summary of HPLC-HRMS criterion for quantification of phenolics in *Thymus lotocephalus* López and Morales extracts.

Compound	Linear range (mg/L)	Intercept	Slope	R^2	LOD (µg/L)	LOQ (µg/L)
Epigallocatechin gallate	3.12-50	-69150	21667	0.9976	228.13	760.46
Caffeic acid	0.05-8	209431	5791726	0.9999	0.95	3.16
Protocatechuic acid	0.05-10	150321	2058784	0.9995	3.92	13.06
Gallic acid	0.1-50	124985	3119019	0.9993	1.38	4.60
p -Coumaric acid	0.5-6	2090	84084	0.9979	43.91	146.38
Luteolin	0.03-91	7840120	-158687	0.9997	0.60	2.00
Quercetin	0.1-3.12	-1347970	9918400	0.9975	0.10	0.33
Catechin	0.8-25	-632768	2024864	0.9994	3.25	10.82
Rosmarinic acid	0.10-80	-2568152	1567304	0.9917	30.11	100.38

Table S3. EE(λ) x I(λ) constants used in the determination of SPF for each wavelength (Sayre et al., 1979)

Wavelength λ (nm)	EE (λ)x I(λ)
290	0.0150
295	0.0817
300	0.2874
305	0.3278
310	0.1864
315	0.0837
320	0.0180

EE (λ): erythemal effect spectrum; I(λ): solar intensity spectrum.
